# Biomechanical study of transsacral-transiliac screw fixation versus lumbopelvic fixation and bilateral triangular fixation for “H”- and “U”-type sacrum fractures with traumatic spondylopelvic dissociation: a finite element analysis study

**DOI:** 10.1186/s13018-021-02581-5

**Published:** 2021-07-03

**Authors:** Ye Peng, Gongzi Zhang, Shuwei Zhang, Xinran Ji, Junwei Li, Chengfei Du, Wen Zhao, Lihai Zhang

**Affiliations:** 1 Department of Orthopaedic Surgery, General Hospital of Chinese People’s Liberation Army, 28 Fu-Xing Road, 100853 Beijing, People’s Republic of China; 2 Tianjin Key Laboratory for Advanced Mechatronic System Design and Intelligent Control, School of Mechanical Engineering, Tianjin University of Technology, Tianjin, People’s Republic of China; 3 Department of Orthopedics, Beijing Aerospace General Hospital, Beijing, People’s Republic of China

**Keywords:** Lumbopelvic fixation, Transsacral-transiliac screw, Biomechanics, Finite element analysis

## Abstract

**Objective:**

To compare the biomechanical stability of transsacral-transiliac screw fixation and lumbopelvic fixation for “H”- and “U”-type sacrum fractures with traumatic spondylopelvic dissociation.

**Methods:**

Finite element models of “H”- and “U”-type sacrum fractures with traumatic spondylopelvic dissociation were created in this study. The models mimicked the standing position of a human. Fixation with transsacral-transiliac screw fixation, lumbopelvic fixation, and bilateral triangular fixation were simulated. Biomechanical tests of instability were performed, and the fracture gap displacement, anteflexion, rotation, and stress distribution after fixation were assessed.

**Results:**

For H-type fractures, the three kinds of fixation ranked by stability were bilateral triangular fixation > lumbopelvic fixation > transsacral-transiliac screw fixation in the vertical and anteflexion directions, bilateral triangular fixation > transsacral-transiliac S1 and S2 screw fixation > lumbopelvic fixation in rotation. The largest displacements in the vertical, anteflexion, and rotational directions were 0.57234 mm, 0.37923 mm, and 0.13076 mm, respectively. For U-type fractures, these kinds of fixation ranked by stability were bilateral triangular fixation > lumbopelvic fixation > transsacral-transiliac S1 and S2 screw fixation > transsacral-transiliac S1 screw fixation in the vertical, anteflexion, and rotational directions. The largest displacements in the vertical, anteflexion, and rotational directions were 0.38296 mm, 0.33976 mm, and 0.05064 mm, respectively.

**Conclusion:**

All these kinds of fixation met the mechanical criteria for clinical applications. The biomechanical analysis showed better bilateral balance with transsacral-transiliac screw fixation. The maximal displacement for these types of fixation was less than 1 mm. Percutaneous transsacral-transiliac screw fixation can be considered the best option among these kinds of fracture fixation.

## Introduction

Traumatic sacrum fractures and spondylopelvic dissociation are two severe injuries that are caused by falling and high-energy vehicle accidents. “U”-type and “H”-type sacrum fractures are the common subtypes of sacrum fractures [1, 2]. The internal fixation methods commonly used to treat these fractures include percutaneous transsacral-transiliac screw fixation, lumbopelvic fixation, and bilateral triangular fixation [3–6]. There is still controversy regarding the best treatment method for these injuries.

In recent years, with the development of Starr-frame closed reduction tools for pelvic fractures, most pelvic fractures can be reduced without open surgery, and the range of indications for percutaneous fixation has expanded [7, 8]. Percutaneous transsacral-transiliac screw fixation is commonly used but technically demanding because it involves a minimally invasive incision with a narrow bone tunnel. König [9] analyzed the bone tunnel anatomy for S1 and S2 screws, and R.O. Sundaram [10] confirmed that two-thirds of males and females have a complete osseous corridor through which a transsacral-transiliac S1 screw can be passed and that the S2 corridor is present in all males but only in 87% of females. Therefore, transsacral-transiliac screw fixation can be used for an increasing number of cases.

Some biomechanical studies have focused on lumbopelvic fixation and transsacral-transiliac screw fixation [11–14]. However, very few biomechanical studies have focused on the differences among transsacral-transiliac screw fixation, lumbopelvic fixation, and bilateral triangular fixation. In this study, we investigated the biomechanical differences among these three kinds of fixation in “H”- and “U”-type traumatic sacrum fractures with spondylopelvic dissociation through 3D finite element analysis (FEA). This study focused on biomechanical stability, and the results provide useful information for clinical decision-making.

## Materials and methods

### Model construction

The geometric properties of the bony tissue of the pelvis and lumbar spine of a healthy 30-year-old female were assessed by a CT scan with a slice thickness of 1 mm, and the imaging data were then converted into a coarse geometric model by using Mimics 17.0 (Materialise Technologies Company, Leuven, Belgium). The lumbar intervertebral disc cartilage, cortical bones, cancellous bones, articular cartilage, fiber rings, and ligaments of the FE model were created based on the geometrical model in Hypermesh 14.0 (Altair Engineering Corp, MI, USA). The geometric model of the lumbar, ilium, and sacrum was then refined with reverse engineering software (Geomagic 11.0, Geomagic Company. Morrisville, USA) and meshed into a finite element (FE) model with FE preprocessing software (Hyper Mesh 14.0, Altair Engineering Company, MI, USA). Based on the structure of the bone, an FE model of the cartilage and ligaments was created according to the anatomical studies [15, 16] (Table 1).

**Table 1:** Material parameters and element types of the model

	Unit type	Elastic modulus	Poisson’s ratio	Thickness/mm
Cortical bone	S4R	12 000	0.3	0.5
Cancellous bone	C3D8R、C3D4	100	0.2	—
Rear structure	C3D8R、C3D4	3 500	0.25	—
Endplate	S4R	2500	0.25	0.5
Articular cartilage	C3D8R	25	0.4	—
Nucleus pulposus	C3D8R	1.0	0.499 9	—
Annulus	C3D8R	4.2	0.45	—
Anterior longitudinal ligament	T3D2	7.8	0.3	—
Posterior longitudinal ligament	T3D2	10	0.3	—
Ligamentum flavum	T3D2	15	0.3	—
Interspinous ligament	T3D2	10	0.3	—
Supraspinous ligament	T3D2	8	0.3	—
Intertransverse ligament	T3D2	10	0.3	—
Joint capsule ligament	T3D2	7.5	0.3	—

The following ligaments were constructed as 3D tension truss elements: iliolumbar ligament (IL), anterior sacroiliac ligament (ASL), interspinous ligament (ISL), long posterior sacroiliac ligament (LPSL), short posterior sacroiliac ligament (SPSL), sacrospinous ligament (SS), sacrotuberous ligament (ST), superior pubic (SP), arcuate pubic (AP), ligamentum flavum (LF), anterior longitudinal ligament (ALL), posterior longitudinal ligament (PLL), interspinous ligament (IL2), supraspinous ligament (SL), ligamenta intertransversaria (LI), and capsular ligament (CL). The regions were attached according to the methods described in previous studies [15, 16]. The entire model had 886554 elements and 241576 nodes.

### Model validation

**Fig. 1:**
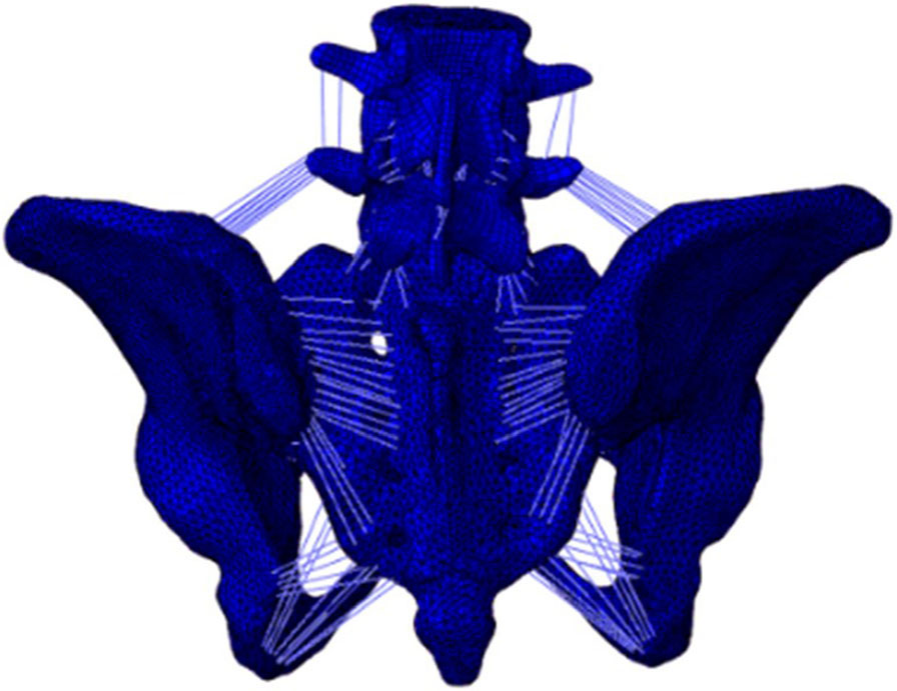
FE model of lumbar vertebrae 4 and 5 and an intact pelvis

The FE model was built based on the methods described in a previous study [17]. The results were validated using in vitro data and the results of other simulation studies [18–20]. According to the in vitro study, the point loads on the ventral surface and dorsal surface of L4 were located in the midsagittal plane of the inferior S1 and superior S2 vertebrae. Five translational loads (294 N) and three rotational moments (42 N*m) (anterior, posterior, superior, inferior, mediolateral, flexion, extension, and axial rotation) were tested. The validation outcomes were satisfactory. The “H”- and “U”-shaped fracture lines and different fixation models of the pelvis were then built based on the intact model according to a previous study [14, 21] (Fig. 1).

### Pelvic FE model of H- and U-type fracture injuries and three kinds of fixation

The model mimicked the standing position of a human. The model was loaded with a 500-N force, and the forces were directed straight downward and toward the center of the L4 superior endplate. Both sides of the ischial tuberosity were restricted to six degrees of freedom.

**Fig. 2:**
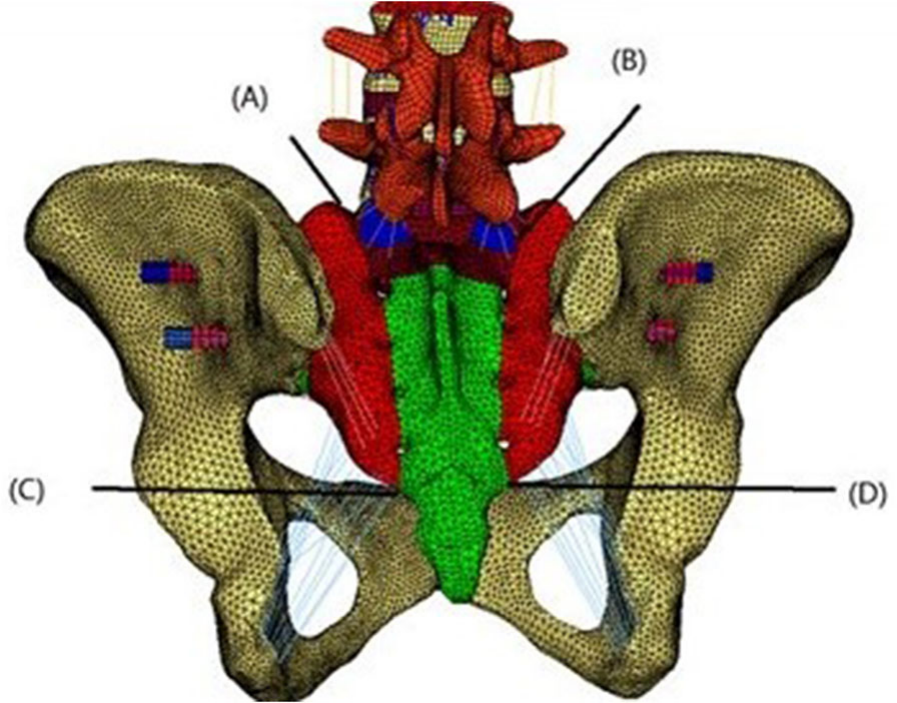
FE model of the H-shape fracture with transsacral-transiliac S1 and S2 screw fixation

**Fig. 3:**
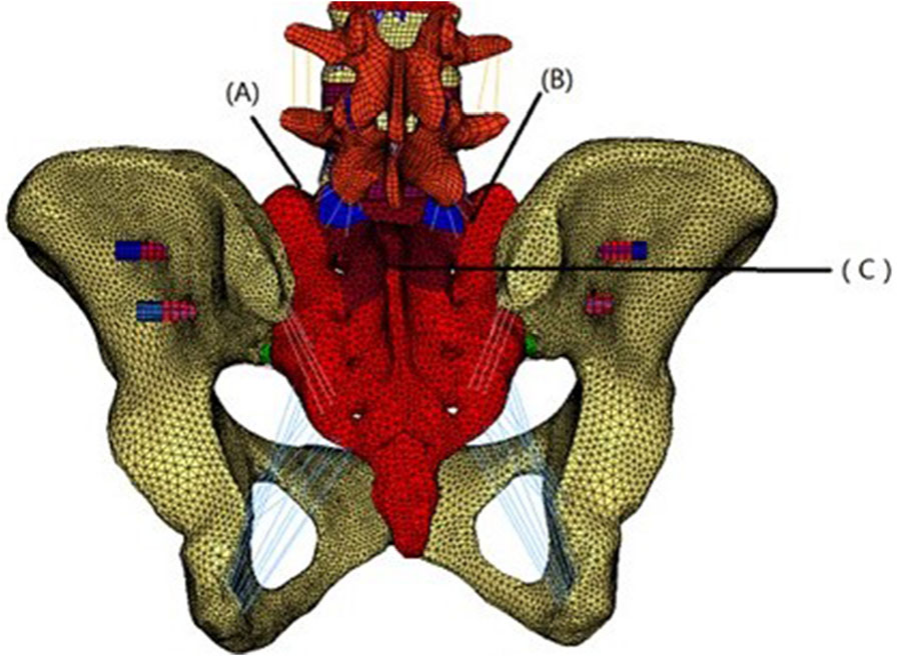
FE model of the U-shaped fracture with transsacral-transiliac S1 and S2 screw fixation

The H-type sacrum fracture involved a fracture line between the two S1 foramina and a vertical fracture line crossing both sides of the sacral foramina (Fig. 2). The U-type sacrum fracture involved a fracture line between the two S2 foramina and a vertical fracture line from both sides of the top of the sacrum to the S2 foramina (Fig. 3). We use 7.3 mm full thread cannulated screw for fixation that will provide strong strength for fixation.

Four points, A to D, were marked on the model of the H-type sacrum fracture. A was located at the top of the fracture line on the left. B was located at the top of the fracture line on the right. C was located at the bottom of the fracture line on the left. D was located at the bottom of the fracture line on the right. After loading, the displacement of the four points was recorded. The displacement values after loading were recorded as A_1_ to D_1_.

Three points, A to C, were marked on the model of the U-type sacrum fracture. A was located at the top of the fracture line on the left. B was located at the top of the fracture line on the right. C was located in the middle of the S2 horizon fracture line. After loading, the displacement of the three points was recorded. The displacement values before and after loading were recorded as A_1_ to C_1_.

We chose the following 4 typical methods of fixation that many clinical studies and biomechanical studies have used: single S1 transsacral-transiliac screw fixation, S1 and S2 transsacral-transiliac screw fixation (transsacral-transiliac S1 and S2 screw), L4-L5 pedicle screw and iliac screw lumbopelvic fixation (lumbopelvic fixation), and S1 transsacral-transiliac screw combinations of L4-L5 pedicle screw and iliac screw lumbopelvic fixation( bilateral triangular fixation). We used single S1 transsacral-transiliac screw fixation for the U-type fractures but not for the H-type fractures.

The stress distribution of internal fixation and displacement of the points on the model were recorded to determine the biomechanical stability and loading situations.

## Results

### H-type sacrum fracture displacement and internal fixation stress distribution

The vertical displacement of the four points of the H-type fracture gap under load ranged from 0.40162 mm to 0.57234 mm with transsacral-transiliac S1 and S2 screw fixation, 0.36754 mm to 0.55696 mm with lumbopelvic fixation (L4–L5 pedicle screw and iliac screw fixation), and 0.09874 mm to 0.55761 mm with bilateral triangular fixation (transsacral-transiliac S1 screw and lumbopelvic fixation) (Table 2). The maximal displacement values at the four points in the 3 models were AA1 with transsacral-transiliac S1 and S2 screw fixation and DD1 with both lumbopelvic fixation and bilateral triangular fixation.

**Table 2:** Vertical load displacement (mm) of the fracture gap for 3 kinds of fixation performed for the H-type fracture

H-type fracture	Displacement (mm)			
	AA1	BB1	CC1	DD1
T-S1S2 fixation	0.57234	0.40162	0.50403	0.52396
Lumbopelvic fixation	0.36754	0.39059	0.50983	0.55696
Bilateral triangular fixation	0.15739	0.09874	0.16289	0.55761

The anteflexion displacement of the four points for the H-type fracture gap under load ranged from 0.31452 mm to 0.37923 mm with transsacral-transiliac S1 and S2 screw fixation, 0.18736 mm to 0.25996 mm with lumbopelvic fixation, and 0.03491 mm to 0.28737 mm with bilateral triangular fixation (Table 3). The maximal displacement values at the four points in the 3 models were AA1 with transsacral-transiliac S1 and S2 screw fixation and DD1 with lumbopelvic fixation and bilateral triangular fixation. The rotational displacement values of these four points of the H-type fracture gap under load ranged from 0.01065 mm to 0.06149 mm with transsacral-transiliac S1 and S2 screw fixation, 0.02566 mm to 0.13076 mm with lumbopelvic fixation, and 0.01128 mm to 0.09121 mm with bilateral triangular fixation (Table 4). The maximal displacement values at the four points in the 3 models were AA1 with transsacral-transiliac S1 and S2 screw fixation and DD1 with lumbopelvic fixation and bilateral triangular fixation.

**Table 3:** Anteflexion displacement (mm) with a load of 7.5 N.m in the standing position

H-type fracture	Displacement (mm)			
	AA1	BB1	CC1	DD1
T-S1S2 fixation	**0.37923**	0.32204	0.31452	0.32229
Lumbopelvic fixation	0.18867	0.18736	0.25769	**0.25996**
Bilateral triangular fixation	0.05174	0.03491	0.27553	**0.28737**

**Table 4:** Rotational displacement (mm) with a load of 7.5 N.m in the standing position

H-type fracture	Displacements (mm)			
	AA1	BB1	CC1	DD1
T-S1S2 fixation	0.03979	0.03056	**0.06149**	0.01065
Lumbopelvic fixation	0.02566	0.10199	0.02888	**0.13076**
bilateral triangular fixation	0.01128	0.01811	0.08688	**0.09121**

**Fig. 4:**
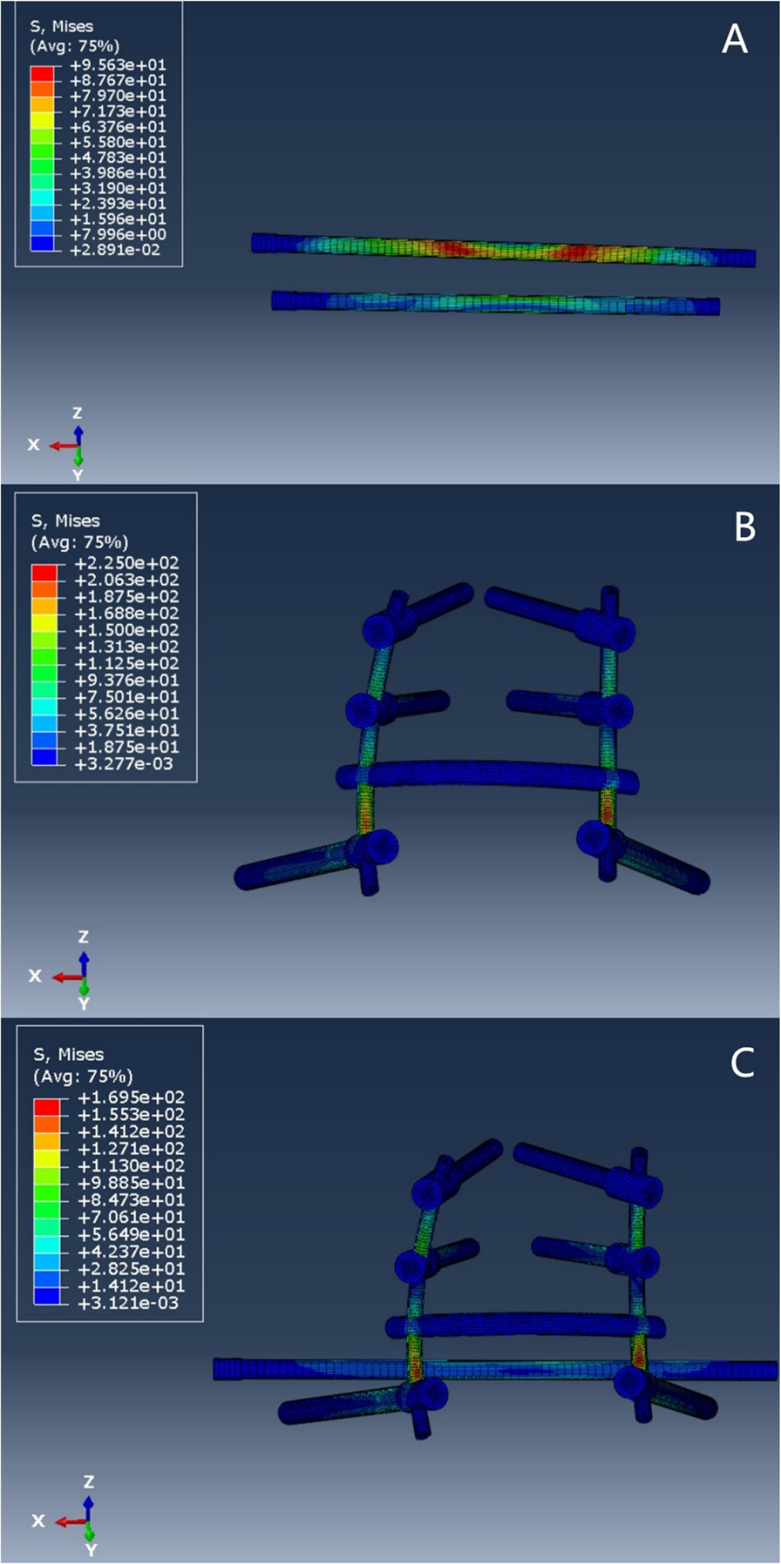
H-type fracture fixation stress distribution

For the H-type sacrum fracture, the areas of maximal stress of internal fixation for the 3 types of internal fixation were the fracture site and the S1 screw with transsacral-transiliac S1 and S2 screw fixation (9.563e+01) and at the bar above the iliac screw with both lumbopelvic fixation (2.250e+02) and bilateral triangular fixation (1.695e+02) Fig. 4.

### U-type sacrum fracture displacement and internal fixation stress distribution

The vertical displacement of the three points for the U-type fracture gap under load ranged from 0.20655 mm to 0.38296 mm with single S1 transsacral-transiliac screw fixation, 0.17605 mm to 0.33581 mm with S1 and S2 transsacral-transiliac screw fixation, 0.14006 mm to 0.27315 mm with lumbopelvic fixation, and 0.02739 mm to 0.06335 mm with bilateral triangular fixation (Table 5). The maximal displacement was C1C2 with transsacral-transiliac S1 fixation and transsacral-transiliac S1 and S2 screw fixation and A1A2 with lumbopelvic fixation and bilateral triangular fixation.

**Table 5:** Vertical load displacement (mm) of the fracture gap for the 4 kinds of fixation for the U-type fracture

U-type fracture	Displacement (mm)		
	A1A2	B1B2	C1C2
Single T-S1 fixation	0.35635	0.20655	**0.38296**
T-S1 S2 fixation	0.31875	0.17605	**0.33581**
Lumbopelvic fixation	**0.27315**	0.20383	0.14006
Bilateral triangular fixation	**0.06335**	0.02739	0.05047

The anteflexion displacement of the three points for the U-type fracture gap under load ranged from 0.17562 mm to 0.33976 mm with single S1 transsacral-transiliac screw fixation, 0.11810 mm to 0.23176 mm with S1 and S2 transsacral-transiliac screw fixation, 0.05764 mm to 0.12509 mm with L4–L5 pedicle screw and iliac screw lumbopelvic fixation, and 0.00691 mm to 0.02362 mm with bilateral triangular fixation (Table 6). The maximal displacement was C1C2 with single T-S1 and transsacral-transiliac S1 and S2 screw fixation and A1A2 with lumbopelvic fixation and bilateral triangular fixation.

**Table 6:** Anteflexion displacement (mm) under a load of 7.5 N.m in the standing position

U-type fracture	Displacement (mm)		
	A1A2	B1B2	C1C2
Single T-S1 fixation	0.24481	0.17562	**0.33976**
T-S1S2 fixation	0.18271	0.11810	**0.23176**
Lumbopelvic fixation	**0.12509**	0.08782	0.05764
Bilateral triangular fixation	**0.02362**	0.00691	0.01640

The rotational displacement of the three points for the U-type fracture gap under load ranged from 0.03034 mm to 0.05064 mm with single S1 transsacral-transiliac screw fixation, 0.02353 mm to 0.04319 mm with S1 and S2 transsacral-transiliac screw fixation, 0.01480 mm to 0.09143 mm with lumbopelvic fixation, and 0.00452 mm to 0.02920 mm with bilateral triangular fixation (Table 7). The maximal displacement was C1C2 with single transsacral-transiliac screw fixation and transsacral-transiliac S1 and S2 screw fixation and A1A2 with lumbopelvic fixation and bilateral triangular fixation.

**Table 7:** Rotational displacement (mm) under a 7.5-N.m load in the standing position

U-type fracture	Displacement (mm)		
	A1A2	B1B2	C1C2
Single T-S1 fixation	0.04347	0.03034	**0.05064**
T-S1S2 fixation	0.04059	0.02353	**0.04319**
Lumbopelvic fixation	0.01480	**0.09143**	0.03368
Bilateral triangular fixation	0.01015	0.00452	**0.02920**

**Fig. 5:**
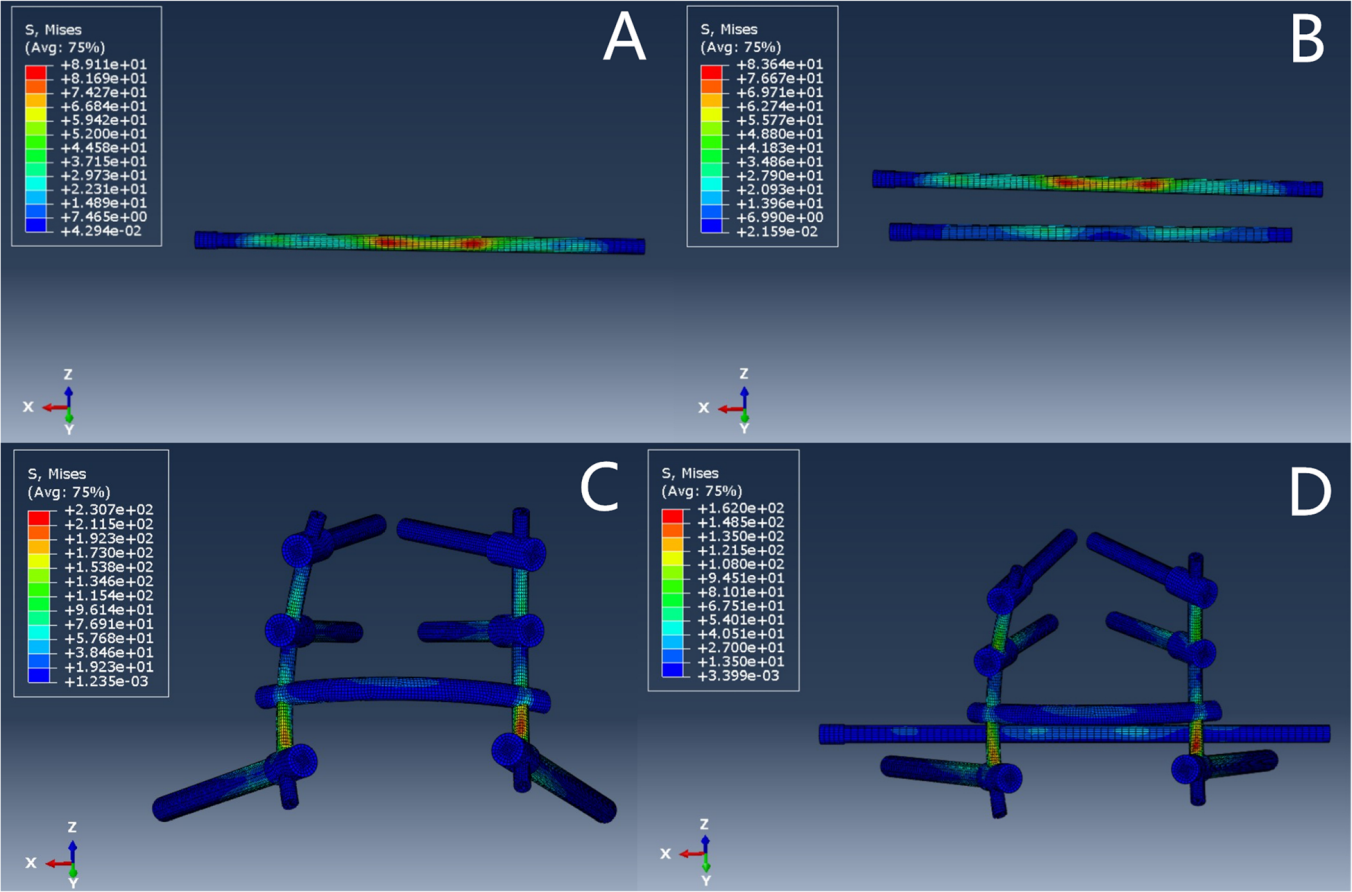
U-type fracture fixation stress distribution

For the U-type sacrum fracture, the areas of maximal stress of internal fixation for the 4 types of internal fixation were the fracture site on the S1 screw with transsacral-transiliac S1 and S2 screw fixation (8.364e+01) and at the bar above the iliac screw with lumbopelvic fixation (2.307e+02), bilateral triangular fixation (1.620e+02), and single S1 fixation (8.911e+01) Fig. 5.

## Discussion

This FEA study aimed to study the biomechanics of internal fixation for “H”- and “U”-type sacrum fractures with traumatic spondylopelvic dissociation. Since the Starr frame is popular, closed reduction with percutaneous fixation can be used to treat an increasing number of displaced pelvic fractures. Routt et al. [3] was the first to perform percutaneous transsacral-transiliac fixation for U-shaped fractures but not for H-shaped fractures. Many studies suggest that percutaneous transsacral-transiliac fixation results in less blood loss, a shorter operative time, and fewer postoperative wound problems than does the traditional method [3, 9, 22]. Lumbopelvic fixation and bilateral triangle fixation provide very strong biomechanical support, which can provide multiplanar stability and allow early postoperative mobilization with full weight bearing [23–25]. However, very few works have compared the biomechanical differences among percutaneous transsacral-transiliac fixation, L4–L5 pedicle screw and iliac screw lumbopelvic fixation, and bilateral triangular fixation. We considered that the biomechanics of percutaneous transsacral-transiliac fixation meet the criteria for clinical applications. In this study, we utilized an FE model to analyze the lumbar spine, pelvis, and internal fixations, and some common fixation constructs were evaluated for “H”- and “U”-type sacrum fractures with traumatic spondylopelvic dissociation.

For H-type fractures, our data showed that all three kinds of internal fixation yield great biomechanical stability in the standing position, and none of the fractures displacement values exceeded 1 mm. In transsacral-transiliac S1 and S2 screw fixation, there was great symmetry of the four points at the fracture sites (difference value less than 0.2 mm). The maximal displacement was at AA1 at the top of the fracture line on the left. In lumbopelvic fixation, there was less displacement at the top of the fracture site (0.36754 mm and 0.39059 mm) and slightly more displacement at the bottom (0.50983 mm and 0.55696 mm), which means that L4–L5 pedicle screw and iliac screw lumbopelvic fixation yields more stability at the top of fractures and great bilateral symmetry. The maximal displacement was at DD1 at the bottom of the fracture line on the right. In the bilateral triangular fixation, there was less displacement (less than 0.2 mm) at points A, B, and C, which means that this kind of fixation is the most stable. The maximal displacement was at DD1 at the bottom of the fracture line on the right. Adding S1 transsacral-transiliac screw fixation can increase the stability at the top of the fracture site. Regarding anteflexion displacement, the results showed that maximal displacement at AA1 with transsacral-transiliac S1 and S2 screw fixation was 0.37923 mm, which is less than 1 mm. This means that lumbopelvic fixation yields more stability than does transsacral-transiliac fixation and that bilateral triangular fixation is the most stable. For rotation displacement, the results showed that the maximal displacement was 0.13076 mm with lumbopelvic fixation. Additionally, the displacement with transsacral-transiliac S1 and S2 screw fixation was, on average, less than that with bilateral triangular fixation, which means that lumbopelvic fixation was more rotationally stable than was transsacral-transiliac screw fixation. The displacement values for all three kinds of fixation were less than 1 mm, which meets the clinical requirements. In summary, for H-type fractures, these three kinds of fixation ranked by stability were bilateral triangular fixation > lumbopelvic fixation > transsacral-transiliac S1 and S2 screw fixation > transsacral-transiliac S1 screw fixation in the vertical and anteflexion directions, bilateral triangular fixation > transsacral-transiliac S1 and S2 screw fixation > lumbopelvic fixation in rotation.

For U-type fractures, our displacement data showed that the three kinds of internal fixation yielded great biomechanical stability in the standing position, and none of the methods had displacement values exceeding 1 mm. In transsacral-transiliac S1 and S2 screw fixation, there was great symmetry for the three points on the fracture sites (difference value less than 0.2 mm). The maximal displacement was at AA1 at the top of the fracture line on the left. In the lumbopelvic fixation, there was less displacement at the top of the fracture site (0.36754 mm and 0.39059 mm) and slightly more displacement at the bottom (0.50983 mm and 0.55696 mm), which means that L4–L5 pedicle screw and iliac screw lumbopelvic fixation yields more stability at the top of fractures and great bilateral symmetry. The maximal displacement was at DD1 at the bottom of the fracture line on the right. In the bilateral triangular fixation, there was less displacement (less than 0.2 mm) at points A, B, and C, which means that this kind of fixation is the most stable. The maximal displacement was at DD1 at the bottom of the fracture line on the right. Adding S1 transsacral-transiliac screw fixation can increase the stability at the top of the fracture site. The maximal stress distribution location of internal fixation for the 4 types internal fixation were the fracture site with S1 screw in transsacral-transiliac S1 and S2 screw fixation (8.364e+01) and at the bar above the iliac screw with lumbopelvic fixation (2.307e+02), bilateral triangular fixation (1.620e+02), and single S1 fixation (8.911e+01). Therefore, single S1 and transsacral-transiliac S1 and S2 screw fixation have more biomechanical symmetry in internal fixations and slightly higher stress concentrations than does lumbopelvic fixation. Lumbopelvic fixation leads to stress being concentrated on the lower part of the bar, which can be decreased slightly by adding transsacral-transiliac S1 screws. In summary, for U-type fractures, these 4 kinds of fixation methods ranked by stability were bilateral triangular fixation > lumbopelvic fixation > transsacral-transiliac S1 and S2 screw fixation > transsacral-transiliac S1 screw fixation in the vertical, anteflexion, and rotation directions.

For U-type and H-type fractures, percutaneous transsacral-transiliac fixations, L4–L5 pedicle screw and iliac screw lumbopelvic fixation and bilateral triangular fixation can offer sufficient biomechanical stability. However, percutaneous transsacral-transiliac fixation yields better symmetry and a slightly higher stress concentration than does lumbopelvic fixation in U-type and H-type fractures. In the clinic, percutaneous transsacral-transiliac fixation leads to fewer complications and greater range of motion in L4–L5–S1 than does lumbopelvic fixation or bilateral triangular fixation. Therefore, we considered that percutaneous screw fixation (transsacral-transiliac S1 and S2 screw fixation for H-type fractures and transsacral-transiliac S1 screw fixation for U-type fractures) are the optimal choices for clinical applications.

There were some limitations of our study. First, it was based on an FE model of the pelvis after surgical fixation, which may not exactly reflect how a biological pelvis would behave. Second, the pelvic model we generated may not be completely representative of all pelvic morphologies and kinematics/dynamics seen in the general population and thus may be an oversimplification. Additionally, this FE model considers the behavior of the pelvis in a static environment, and our results may differ in more dynamic settings. Additionally, some muscle and ligaments of pelvic and lumbar constructs exist that were not considered and may yield differing results. However, we feel that our study offers a good representation of the available constructs utilized to typically address H- and U-type fractures, and the FE model utilized accurately represents the clinical dilemma at present. Nevertheless, additional studies are warranted to evaluate the applicability of our findings in clinical practice.

## Conclusion

For orthopedics, more and more studies can be done through translational medical [26, 27]. In this study, we analyzed the biomechanics of “H”- and “U”-type sacrum fractures with traumatic spondylopelvic dissociation after percutaneous fixation and S1 and S2 transsacral-transiliac screw fixation, L4–L5 pedicle screw fixation and iliac screw lumbopelvic fixation. The biomechanical stability in the standing position was assessed by the displacement, anteflexion, rotation, and stress distribution after fixation. Transsacral-transiliac screw fixation was quite sufficient for U- and H-type fractures and yielded great symmetry and met the clinical displacement requirements. Bilateral triangular fixation was the strongest fixation and can be used for some severely unstable cases. These FEA results can help surgeons understand the relevance of fixation and biomechanical changes and can guide clinical treatment.

## Abbreviations

FE: Finite element
FEA: Finite element analysis
